# The crystal structure of (*E*)-2-ethyl-*N*-(4-nitro­benzyl­idene)aniline: three-dimensional supra­molecular assembly mediated by C—H⋯O hydrogen bonds and nitro⋯π(arene) inter­actions

**DOI:** 10.1107/S2056989018009544

**Published:** 2018-07-10

**Authors:** Marisiddaiah Girisha, Belakavadi K. Sagar, Hemmige S. Yathirajan, Ravindranath S. Rathore, Christopher Glidewell

**Affiliations:** a Department of Studies in Chemistry, University of Mysore, Manasagangotri, Mysuru 570 006, India; b Centre for Biological Sciences (Bioinformatics), School of Earth, Biological and Environmental Sciences, Central University of South Bihar, Patna 800 014, India; c School of Chemistry, University of St Andrews, St Andrews, Fife KY16 9ST, UK

**Keywords:** Schiff bases, crystal structure, disorder, mol­ecular conformation, hydrogen bonding, nitro⋯π(arene) inter­actions, supra­molecular assembly

## Abstract

In the mol­ecule of the title compound, C_15_H_14_N_2_O_2_, the 2-ethyl­phenyl group is disordered over two sets of atomic sites having occupancies of 0.515 (19) and 0.485 (19), and the dihedral angle between the two partial-occupancy aryl rings is 6(2)°. A combination of C—H⋯O hydrogen bonds and nitro⋯π(arene) inter­actions links the mol­ecules into a continuous three-dimensional framework structure. Comparisons are made with the structures of some related compounds.

## Abstract 2

The 2-ethyl­phenyl group in the title compound is disordered over two sets of atomic sites and the mol­ecules are linked into a three-dimensional array by a combination of C—H⋯O hydrogen bonds and nitro⋯π(arene) inter­actions.

## Chemical context   

**Figure 1:**
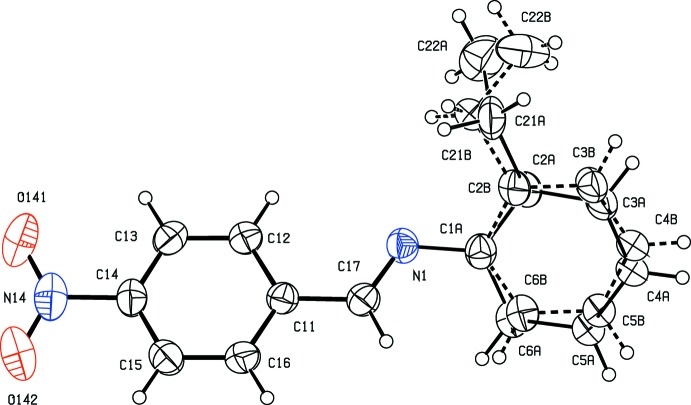
The mol­ecular structure of compound (I) showing the atom-labelling scheme. Displacement ellipsoids are drawn at the 30% probability level, and for the disordered 2-ethyl­phenyl group, the major component is drawn using solid lines and the minor component is drawn using dashed lines.

Schiff bases exhibit a very wide range of biological activities (da Silva *et al.*, 2011 ▸) and are also of inter­est because of their photochromic and thermochromic properties (Hadjoudis & Mavridis, 2004 ▸; Minkin *et al.*, 2011 ▸). In view of the general importance of Schiff bases, and in a continuation of our own structural study of compounds of this type (Girisha *et al.*, 2017 ▸, 2018 ▸) we report here the mol­ecular and supra­moleuclar structure of (*E*)-2-ethyl-*N*-(4-nitro­benzyl­idene)aniline (I) (Fig. 1 ▸), where the ethyl group turns out to be disordered over two sets of atomic sites and where the mol­ecules are linked into a three-dimensional supra­molecular array.
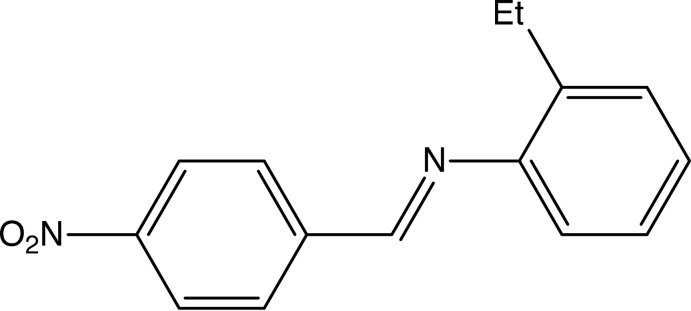


## Structural commentary   

The 2-ethyl­phenyl group in compound (I) is disordered over two sets of atomic sites having occupancies of 0.515 (19) and 0.485 (19) and it is possible that the ethyl group is simply making full use of an available space within the structure: the dihedral angle between the two components of the disordered aryl ring is 6(2)°. The nitro group is almost coplanar with the adjacent aryl ring, with a dihedral angle of only 8.3 (2)° between these two units; on the other hand, the dihedral angles between the nitrated aryl ring and the major and minor components of the disordered ring are 36.7 (10)° and 42.6 (11)°, respectively. The mol­ecules of (I) are therefore conformationally chiral but, in the absence of significant resonant scattering, it was not possible to determine the absolute configuration of the mol­ecules in the crystal selected for data collection. It is reasonable to assume that, in solution, the two conformational enanti­omers will be in rapid equilibrium.

The conformational behaviour of compound (I) may be compared with that of some closely related compounds. In (*E*)-*N*-(4-nitro­benzyl­idene)-2,3-di­methyl­aniline, (II) (Tariq *et al.*, 2010 ▸), and (*E*)-*N*-(4-nitro­benzyl­idene)-3,4-di­meth­oxy­aniline, (III) (Akkurt *et al.*, 2008 ▸), the dihedral angles between the two aryl rings are 24.52 (5) and 29.52 (8)°, respectively. By contrast, in (*E*)-*N*-(4-nitro­benzyl­idene)-2-hy­droxy­aniline), (IV) (Madhuprasad *et al.*, 2014 ▸), and (E)-*N*-(4-chloro­benzyl­idene)-2-hy­droxy­aniline, (V) (Girisha *et al.*, 2018 ▸), the dihedral angles between the rings are 0.52° [the atomic coordinates retrieved from the CSD (Groom *et al.*, 2016 ▸) carry no s.u.s] and 3.31 (9)° respectively, reflecting the influence of the intra­molecular O—H⋯N hydrogen bonds in these two compounds.

## Supra­molecular features   

**Figure 2:**
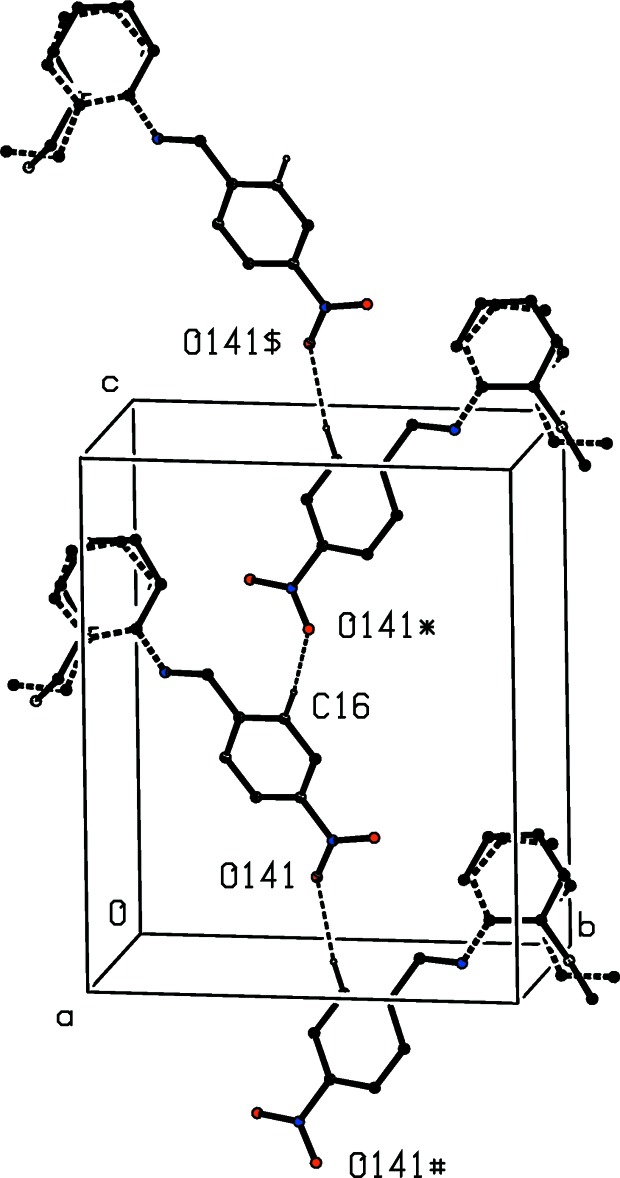
Part of the crystal structure of compound (I) showing the formation of a *C*(6) hydrogen-bonded chain along [001]. For the sake of clarity, the H atoms not involved in the motif shown have been omitted. The atoms marked with an asterisk (*), a hash (#) or a dollar sign ($) are at the symmetry positions (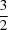 − *x*, 1 − *y*, 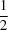 + *z*), (*x*, *y*, 1 + *z*) and (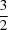 − *x*, 1 − *y*, −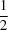 + *z*), respectively. For the disordered 2-ethyl­phenyl group, the major component is drawn using solid lines and the minor component is drawn using dashed lines.

**Figure 3:**
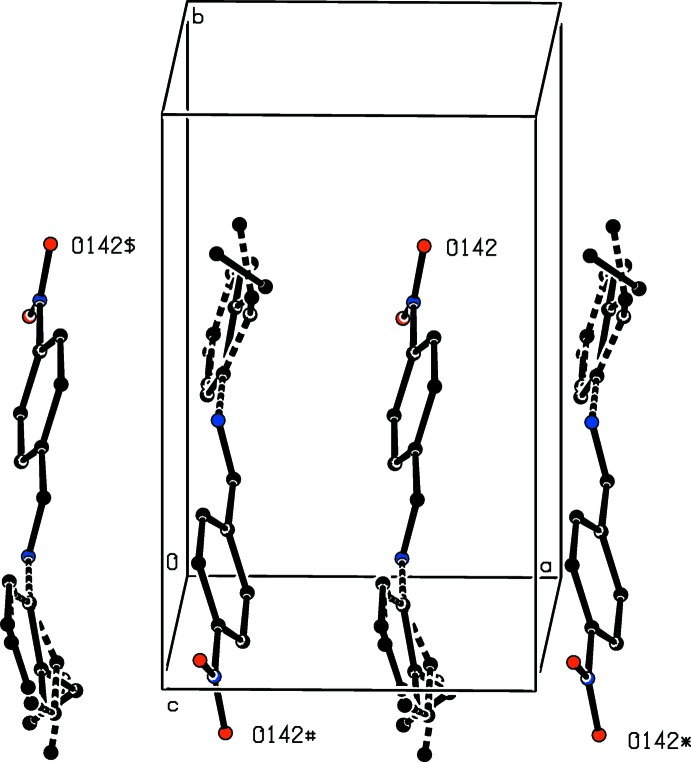
Part of the crystal structure of compound (I) showing the formation of a chain along [100] built from nitro⋯π(arene) inter­actions. For the sake of clarity, the H atoms have all been omitted. The atoms marked with an asterisk (*), a hash (#) or a dollar sign ($) are at the symmetry positions (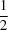 + *x*, 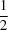 − *y*, 1 − *z*), (−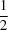 + *x*, 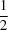 − *y*, 1 − *z*) and (−1 + *x*, *y*, *z*), respectively. For the disordered 2-ethyl­phenyl group, the major component is drawn using solid lines and the minor component is drawn using dashed lines.

**Figure 4:**
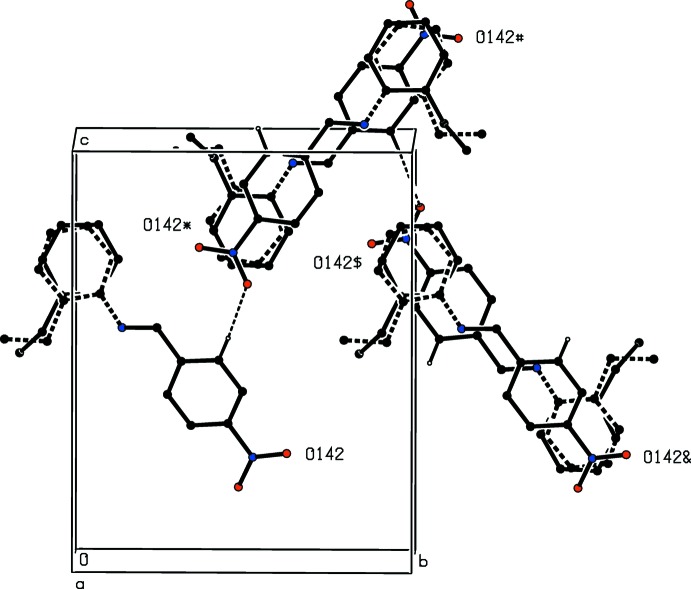
Part of the crystal structure of compound (I) showing the formation of a chain parallel to the [010] direction built from alternating C—H⋯O hydrogen bonds and nitro⋯π(arene) inter­actions. For the sake of clarity, the H atoms not involved in the motif shown have been omitted. The atoms marked with an asterisk (*), a hash (#), a dollar sign ($) or an ampersand (&) are at the symmetry positions (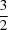 − *x*, 1 − *y*, 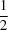 + *z*), (1 − *x*, 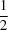 + *y*, 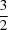 − *z*), (−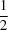 + *x*, 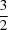 − *y*, 1 − *z*) and (*x*, 1 + *y*, *z*), respectively. For the disordered 2-ethyl­phenyl group, the major component is drawn using solid lines and the minor component is drawn using dashed lines.

The supra­molecular assembly depends upon a combination of one C—H⋯O hydrogen bond (Table 1 ▸) and three N—O⋯π(arene) inter­actions (Kaafarani *et al.*, 2003 ▸; Báuza *et al.*, 2016 ▸) (Table 2 ▸), and the three-dimensional assembly can readily be analysed in terms of three one-dimensional substructures (Ferguson *et al.*, 1998*a* ▸,*b* ▸; Gregson *et al.*, 2000 ▸). Thus, the action of the C—H⋯O hydrogen bond alone is to link mol­ecules related by the 2_1_ screw axis along (0.75, 0.5, *z*) into a *C*(6) chain running parallel to the [001] direction (Fig. 2 ▸). The action of the two nitro⋯π(arene) inter­actions links mol­ecules related by the 2_1_ screw axis along (*x*, 0.25, 0.5) into a chain running parallel to the [100] direction (Fig. 3 ▸), while the combined action of the hydrogen bond and the nitro⋯π(arene) inter­actions links the mol­ecules into a chain running parallel to the [010] direction (Fig. 4 ▸). The combination of chain motifs parallel to the [100], [010] and [001] directions then generates a continuous three-dimensional assembly.

## Database survey   

It is of inter­est to briefly compare the three-dimensional supra­molecular assembly in compound (I), with the patterns of aggregation found in related compounds (II)–(V). In compound (II), two independent aromatic π–π stacking inter­actions combine to link the mol­ecules into chains (Tariq *et al.*, 2010 ▸). The structure of compound (III) (Akkurt *et al.*, 2008 ▸) contains three short C—H⋯O contacts, but two of these involve an H atom in a methyl group, while for the third the C—H⋯O angle is only 131°, so that none of these contacts is likely to be structurally significant (Wood *et al.*, 2009 ▸). The mol­ecules of compound (IV) (Madhuprasad *et al.*, 2014 ▸) are linked into centrosymmetric dimers by inversion-related O—H⋯O hydrogen bonds, while those of compound (V) are linked into a three-dimensional framework structure by a combination of C—H⋯O and C—H⋯π(arene) hydrogen bonds and an aromatic π–π stacking inter­action (Girisha *et al.*, 2018 ▸).

Other Schiff bases which are derived from nitro­benzaldehydes and whose structures have been reported recently include *N*-(2-nitro­benzyl­idene)aniline (Naveen *et al.*, 2006 ▸), 4-meth­oxy-*N*-(2-nitro­benzyl­idene)aniline (Ren & Jian, 2008 ▸), 2,3-dimethyl-*N*-(2-nitro­benzyl­idene)aniline (Tahir *et al.*, 2010 ▸) and 2-fluoro-*N*-(3-nitro­benzyl­idene)-5-(tri­fluoro­meth­yl)aniline (Yang *et al.*, 2007 ▸).

## Synthesis and crystallization   

Solutions of 2-ethyl­aniline (100 mg, 0.826 mmol) and 4-nitro­benzaldehyde (124 mg, 0.826 mmol), each in ethanol (15 ml). were mixed and a catalytic amount of glacial acetic acid was added. The resulting mixture was heated under reflux for 3 h, when completion of the reaction was confirmed using thin layer chromatography. The solid product was collected by filtration and recrystallized from aceto­nitrile to give crystals of (I) suitable for single crystal X-ray diffraction; yield 150mg, 0.590 mmol, 71%; m.p. 369–373 K.

## Refinement   

It was apparent from an early stage in the refinement that the methyl group of the ethyl substituent was disordered over two sets of atomic sites having unequal occupancies, and satisfactory resolution of the disorder required a model in which the whole 2-ethyl­phenyl unit was disordered over two sets of atomic sites. For the minor disorder component, the bonded distances and the 1,3 non-bonded distances were restrained to be the same as the corresponding distances in the major disorder component, subject to s.u. values of 0.01 and 0.02 Å, respectively. In addition, the anisotropic displacement parameters for the corresponding pairs of C atoms in the disordered ring were constrained to be identical. All H atoms apart from those in the ethyl unit were located in difference maps and then treated as riding atoms with C—H 0.93 Å and *U*_iso_(H) = 1.2*U*_eq_(C); the H atoms of the ethyl unit were included in calculated positions with C—H distances of 0.96 Å (CH_3_) or 0.97 Å (CH_2_) and with *U*_iso_(H) = *kU*_eq_(C), where *k* = 1.5 for the methyl groups, which were permitted to rotate but not to tilt, and 1.2 for the CH_2_ groups. Subject to these conditions, the occupancies of the two disorder components refined to 0.515 (19) and 0.485 (19). Although the coverage of Friedel pairs was 98%, it was not possible to determine the absolute configuration of the mol­ecules in the crystal selected for study, as the value of the Flack *x* parameter (Flack, 1983 ▸), calculated using 484 quotients of the type [(*I* ^+^) − (*I* ^−^)]/[(*I* ^+ ^)+(*I* ^−^)] (Parsons *et al.*, 2013 ▸), was −0.5 (7), and value calculated for the Hooft *y* parameter (Hooft *et al.*, 2008 ▸) was −0.4 (7). Crystal data, data collection and structure refinement details are summarized in Table 3 ▸.

## Supplementary Material

Crystal structure: contains datablock(s) global, I. DOI: 10.1107/S2056989018009544/zl2733sup1.cif

Structure factors: contains datablock(s) I. DOI: 10.1107/S2056989018009544/zl2733Isup3.hkl

Supporting information file. DOI: 10.1107/S2056989018009544/zl2733Isup3.cml

CCDC reference: 1853291

Additional supporting information:  crystallographic information; 3D view; checkCIF report

## Figures and Tables

**Table 1:** Hydrogen-bond geometry (Å, °)

*D*—H⋯*A*	*D*—H	H⋯*A*	*D*⋯*A*	*D*—H⋯*A*
C16—H16⋯O141^i^	0.93	2.54	3.456 (5)	167

Symmetry code: (i) 
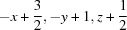
.

**Table 2:** Parameters (Å, °) for nitro⋯π(arene) inter­actions *Cg*1 and *Cg*2 are the centroids of the C1*A*–C6*A* and C1*B*–C6*B* rings, respectively.

N—O⋯*Cg*	N—O	O⋯*Cg*	N⋯*Cg*	N—O⋯*Cg*
N14—O141⋯*Cg*1^i^	1.215 (4)	3.88 (2)	3.91 (2)	82.5 (3)
N14—O141⋯*Cg*2^i^	1.215 (4)	3.82 (2)	3.79 (2)	79.4 (3)
N14—O142⋯*Cg*1^ii^	1.220 (4)	3.97 (2)	3.85 (2)	75.1 (3)

Symmetry codes: (i) −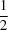 + *x*, 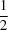 − *y*, 1 − *z*; (ii) 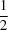 + *x*, 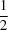 − *y*, 1 − *z*.

**Table 3:** Experimental details

Crystal data	
Chemical formula	C_15_H_14_N_2_O_2_
*M*_r_	254.28
Crystal system, space group	Orthorhombic, *P*2_1_2_1_2_1_
Temperature (K)	296
*a*, *b*, *c* (Å)	7.6419 (7), 11.8889 (13), 14.8082 (16)
*V* (Å^3^)	1345.4 (2)
*Z*	4
Radiation type	Mo *K*α
μ (mm^−1^)	0.09
Crystal size (mm)	0.15 × 0.10 × 0.10

Data collection	
Diffractometer	Bruker APEXII CCD
Absorption correction	Multi-scan (*SADABS*; Bruker, 2012 ▸)
*T*_min_, *T*_max_	0.841, 0.992
No. of measured, independent and observed [*I* > 2σ(*I*)] reflections	20309, 2535, 1401
*R*_int_	0.055
(sin θ/λ)_max_ (Å^−1^)	0.612

Refinement	
*R*[*F* ^2^ > 2σ(*F* ^2^)], *wR*(*F* ^2^), *S*	0.040, 0.120, 1.04
No. of reflections	2535
No. of parameters	209
No. of restraints	17
H-atom treatment	H-atom parameters constrained
Δρ_max_, Δρ_min_ (e Å^−3^)	0.11, −0.11

Computer programs: *APEX2* and *SAINT* (Bruker, 2012 ▸), *SHELXS97* (Sheldrick, 2008 ▸), *SHELXL2014* (Sheldrick, 2015 ▸) and *PLATON* (Spek, 2009 ▸).

## supplementary crystallographic information

### Crystal data

**Table:**

C_15_H_14_N_2_O_2_	*D*_x_ = 1.255 Mg m^−^^3^
*M**_r_* = 254.28	Mo *K*α radiation, λ = 0.71073 Å
Orthorhombic, *P*2_1_2_1_2_1_	Cell parameters from 2535 reflections
*a* = 7.6419 (7) Å	θ = 2.2–25.8°
*b* = 11.8889 (13) Å	µ = 0.09 mm^−^^1^
*c* = 14.8082 (16) Å	*T* = 296 K
*V* = 1345.4 (2) Å^3^	Block, colourless
*Z* = 4	0.15 × 0.10 × 0.10 mm
*F*(000) = 536	

### Data collection

**Table:**

Bruker APEXII CCD diffractometer	2535 independent reflections
Radiation source: fine focus sealed tube	1401 reflections with *I* > 2σ(*I*)
Graphite monochromator	*R*_int_ = 0.055
φ and ω scans	θ_max_ = 25.8°, θ_min_ = 2.2°
Absorption correction: multi-scan (SADABS; Bruker, 2012)	*h* = −6→9
*T*_min_ = 0.841, *T*_max_ = 0.992	*k* = −14→14
20309 measured reflections	*l* = −17→17

### Refinement

**Table:**

Refinement on *F*^2^	Hydrogen site location: inferred from neighbouring sites
Least-squares matrix: full	H-atom parameters constrained
*R*[*F*^2^ > 2σ(*F*^2^)] = 0.040	*w* = 1/[σ^2^(*F*_o_^2^) + (0.0486*P*)^2^ + 0.1129*P*] where *P* = (*F*_o_^2^ + 2*F*_c_^2^)/3
*wR*(*F*^2^) = 0.120	(Δ/σ)_max_ < 0.001
*S* = 1.04	Δρ_max_ = 0.11 e Å^−^^3^
2535 reflections	Δρ_min_ = −0.11 e Å^−^^3^
209 parameters	Extinction correction: SHELXL, Fc^*^=kFc[1+0.001xFc^2^λ^3^/sin(2θ)]^-1/4^
17 restraints	Extinction coefficient: 0.009 (3)

### Special details

**Table:**

Geometry. All esds (except the esd in the dihedral angle between two l.s. planes) are estimated using the full covariance matrix. The cell esds are taken into account individually in the estimation of esds in distances, angles and torsion angles; correlations between esds in cell parameters are only used when they are defined by crystal symmetry. An approximate (isotropic) treatment of cell esds is used for estimating esds involving l.s. planes.

### Fractional atomic coordinates and isotropic or equivalent isotropic displacement parameters (Å^2^)

**Table:**

	*x*	*y*	*z*	*U*_iso_*/*U*_eq_	Occ. (<1)
N1	0.6122 (4)	0.1423 (2)	0.56000 (17)	0.0657 (8)	
C17	0.6533 (5)	0.2454 (3)	0.5623 (2)	0.0643 (9)	
H17	0.6916	0.2765	0.6164	0.077*	
C11	0.6423 (4)	0.3173 (3)	0.4820 (2)	0.0582 (9)	
C12	0.5825 (5)	0.2760 (3)	0.3999 (2)	0.0651 (10)	
H12	0.5473	0.2014	0.3951	0.078*	
C13	0.5754 (5)	0.3451 (3)	0.3261 (2)	0.0689 (10)	
H13	0.5353	0.3177	0.2710	0.083*	
C14	0.6277 (5)	0.4549 (3)	0.3339 (2)	0.0659 (9)	
C15	0.6878 (5)	0.4979 (3)	0.4132 (3)	0.0744 (11)	
H15	0.7234	0.5725	0.4171	0.089*	
C16	0.6949 (5)	0.4281 (3)	0.4880 (2)	0.0704 (10)	
H16	0.7355	0.4562	0.5427	0.084*	
N14	0.6223 (5)	0.5279 (4)	0.2537 (3)	0.0934 (11)	
O141	0.5892 (5)	0.4855 (3)	0.1810 (2)	0.1275 (13)	
O142	0.6516 (5)	0.6280 (3)	0.2643 (2)	0.1273 (13)	
C1A	0.6204 (4)	0.0777 (3)	0.6407 (2)	0.0618 (9)	0.515 (19)
C2A	0.652 (3)	−0.0381 (8)	0.6399 (9)	0.060 (3)	0.515 (19)
C21A	0.739 (2)	−0.0913 (13)	0.5561 (11)	0.089 (6)	0.515 (19)
H21A	0.7893	−0.0320	0.5192	0.107*	0.515 (19)
H21B	0.8340	−0.1398	0.5757	0.107*	0.515 (19)
C22A	0.612 (2)	−0.1593 (16)	0.4994 (10)	0.121 (6)	0.515 (19)
H22A	0.5162	−0.1121	0.4810	0.181*	0.515 (19)
H22B	0.5675	−0.2210	0.5344	0.181*	0.515 (19)
H22C	0.6707	−0.1878	0.4469	0.181*	0.515 (19)
C3A	0.664 (6)	−0.0915 (16)	0.7237 (12)	0.073 (3)	0.515 (19)
H3A	0.6967	−0.1668	0.7255	0.087*	0.515 (19)
C4A	0.628 (5)	−0.0372 (17)	0.8034 (10)	0.072 (4)	0.515 (19)
H4A	0.6420	−0.0747	0.8580	0.087*	0.515 (19)
C5A	0.573 (8)	0.072 (2)	0.8022 (10)	0.075 (3)	0.515 (19)
H5A	0.5359	0.1075	0.8550	0.090*	0.515 (19)
C6A	0.572 (13)	0.130 (3)	0.7204 (13)	0.0739 (19)	0.515 (19)
H6A	0.5383	0.2049	0.7193	0.089*	0.515 (19)
C1B	0.6204 (4)	0.0777 (3)	0.6407 (2)	0.0618 (9)	0.485 (19)
C2B	0.693 (3)	−0.0285 (9)	0.6285 (9)	0.060 (3)	0.485 (19)
C21B	0.704 (3)	−0.0772 (10)	0.5319 (10)	0.079 (6)	0.485 (19)
H21C	0.6174	−0.0412	0.4936	0.095*	0.485 (19)
H21D	0.8188	−0.0627	0.5065	0.095*	0.485 (19)
C22B	0.671 (4)	−0.2034 (12)	0.5351 (15)	0.179 (11)	0.485 (19)
H22D	0.6559	−0.2315	0.4748	0.268*	0.485 (19)
H22E	0.5664	−0.2180	0.5694	0.268*	0.485 (19)
H22F	0.7682	−0.2403	0.5631	0.268*	0.485 (19)
C3B	0.691 (6)	−0.1024 (17)	0.7018 (13)	0.073 (3)	0.485 (19)
H3B	0.7289	−0.1762	0.6937	0.087*	0.485 (19)
C4B	0.635 (5)	−0.0684 (17)	0.7854 (11)	0.072 (4)	0.485 (19)
H4B	0.6331	−0.1195	0.8329	0.087*	0.485 (19)
C5B	0.583 (9)	0.040 (2)	0.7992 (11)	0.075 (3)	0.485 (19)
H5B	0.5538	0.0648	0.8569	0.090*	0.485 (19)
C6B	0.574 (14)	0.113 (3)	0.7262 (14)	0.0739 (19)	0.485 (19)
H6B	0.5364	0.1871	0.7349	0.089*	0.485 (19)

### Atomic displacement parameters (Å^2^)

**Table:**

	*U*^11^	*U*^22^	*U*^33^	*U*^12^	*U*^13^	*U*^23^
N1	0.0710 (19)	0.0625 (19)	0.0635 (18)	−0.0032 (15)	−0.0085 (15)	0.0061 (15)
C17	0.069 (2)	0.065 (2)	0.059 (2)	0.000 (2)	−0.0101 (19)	−0.0022 (19)
C11	0.059 (2)	0.058 (2)	0.057 (2)	0.0020 (17)	−0.0041 (17)	−0.0012 (17)
C12	0.073 (2)	0.062 (2)	0.061 (2)	−0.0010 (17)	−0.0050 (19)	−0.0024 (18)
C13	0.072 (2)	0.080 (3)	0.054 (2)	0.006 (2)	0.0016 (19)	−0.0050 (19)
C14	0.064 (2)	0.072 (2)	0.062 (2)	0.0094 (19)	0.0053 (18)	0.0149 (19)
C15	0.081 (3)	0.059 (2)	0.082 (3)	−0.001 (2)	0.002 (2)	0.004 (2)
C16	0.082 (3)	0.066 (2)	0.063 (2)	0.001 (2)	−0.0072 (19)	−0.0086 (19)
N14	0.085 (2)	0.109 (3)	0.085 (3)	0.009 (2)	0.008 (2)	0.033 (3)
O141	0.148 (3)	0.165 (3)	0.069 (2)	−0.008 (2)	−0.001 (2)	0.032 (2)
O142	0.146 (3)	0.101 (2)	0.135 (3)	−0.002 (2)	0.000 (2)	0.053 (2)
C1A	0.060 (2)	0.065 (2)	0.060 (2)	−0.0052 (19)	−0.0073 (17)	0.0066 (18)
C2A	0.049 (10)	0.060 (3)	0.070 (4)	−0.021 (4)	0.019 (4)	0.008 (3)
C21A	0.120 (11)	0.079 (10)	0.067 (9)	−0.012 (7)	−0.018 (7)	0.030 (7)
C22A	0.131 (11)	0.132 (14)	0.100 (9)	−0.048 (10)	−0.002 (7)	−0.009 (9)
C3A	0.086 (12)	0.057 (4)	0.075 (7)	0.007 (3)	0.026 (10)	0.011 (5)
C4A	0.105 (4)	0.049 (9)	0.064 (5)	0.003 (9)	0.002 (7)	−0.001 (6)
C5A	0.102 (7)	0.059 (12)	0.064 (3)	0.006 (14)	0.003 (2)	0.002 (4)
C6A	0.086 (3)	0.066 (7)	0.070 (3)	0.004 (10)	−0.004 (6)	0.009 (3)
C1B	0.060 (2)	0.065 (2)	0.060 (2)	−0.0052 (19)	−0.0073 (17)	0.0066 (18)
C2B	0.049 (10)	0.060 (3)	0.070 (4)	−0.021 (4)	0.019 (4)	0.008 (3)
C21B	0.123 (12)	0.050 (6)	0.065 (9)	0.004 (7)	−0.002 (8)	0.001 (7)
C22B	0.30 (3)	0.085 (10)	0.147 (17)	−0.007 (13)	0.039 (17)	−0.044 (10)
C3B	0.086 (12)	0.057 (4)	0.075 (7)	0.007 (3)	0.026 (10)	0.011 (5)
C4B	0.105 (4)	0.049 (9)	0.064 (5)	0.003 (9)	0.002 (7)	−0.001 (6)
C5B	0.102 (7)	0.059 (12)	0.064 (3)	0.006 (14)	0.003 (2)	0.002 (4)
C6B	0.086 (3)	0.066 (7)	0.070 (3)	0.004 (10)	−0.004 (6)	0.009 (3)

### Geometric parameters (Å, º)

**Table:**

N1—C17	1.266 (4)	C22A—H22B	0.9600
N1—C1A	1.422 (4)	C22A—H22C	0.9600
C17—C11	1.466 (4)	C3A—C4A	1.372 (10)
C17—H17	0.9300	C3A—H3A	0.9300
C11—C16	1.381 (5)	C4A—C5A	1.370 (9)
C11—C12	1.387 (4)	C4A—H4A	0.9300
C12—C13	1.369 (4)	C5A—C6A	1.390 (8)
C12—H12	0.9300	C5A—H5A	0.9300
C13—C14	1.369 (5)	C6A—H6A	0.9300
C13—H13	0.9300	C2B—C3B	1.397 (9)
C14—C15	1.361 (5)	C2B—C21B	1.547 (11)
C14—N14	1.471 (5)	C21B—C22B	1.523 (13)
C15—C16	1.385 (5)	C21B—H21C	0.9700
C15—H15	0.9300	C21B—H21D	0.9700
C16—H16	0.9300	C22B—H22D	0.9600
N14—O141	1.215 (4)	C22B—H22E	0.9600
N14—O142	1.220 (4)	C22B—H22F	0.9600
C1A—C6A	1.383 (8)	C3B—C4B	1.370 (10)
C1A—C2A	1.398 (8)	C3B—H3B	0.9300
C2A—C3A	1.397 (9)	C4B—C5B	1.368 (9)
C2A—C21A	1.545 (11)	C4B—H4B	0.9300
C21A—C22A	1.520 (13)	C5B—C6B	1.391 (9)
C21A—H21A	0.9700	C5B—H5B	0.9300
C21A—H21B	0.9700	C6B—H6B	0.9300
C22A—H22A	0.9600		
			
C17—N1—C1A	119.3 (3)	H22A—C22A—H22B	109.5
N1—C17—C11	121.9 (3)	C21A—C22A—H22C	109.5
N1—C17—H17	119.1	H22A—C22A—H22C	109.5
C11—C17—H17	119.1	H22B—C22A—H22C	109.5
C16—C11—C12	119.3 (3)	C4A—C3A—C2A	122.5 (9)
C16—C11—C17	119.1 (3)	C4A—C3A—H3A	118.7
C12—C11—C17	121.6 (3)	C2A—C3A—H3A	118.7
C13—C12—C11	120.0 (3)	C5A—C4A—C3A	119.8 (10)
C13—C12—H12	120.0	C5A—C4A—H4A	120.1
C11—C12—H12	120.0	C3A—C4A—H4A	120.1
C12—C13—C14	119.5 (3)	C4A—C5A—C6A	118.6 (9)
C12—C13—H13	120.2	C4A—C5A—H5A	120.7
C14—C13—H13	120.2	C6A—C5A—H5A	120.7
C15—C14—C13	121.9 (3)	C1A—C6A—C5A	121.6 (9)
C15—C14—N14	118.9 (4)	C1A—C6A—H6A	119.2
C13—C14—N14	119.1 (4)	C5A—C6A—H6A	119.2
C14—C15—C16	118.6 (3)	C3B—C2B—C21B	118.9 (11)
C14—C15—H15	120.7	C22B—C21B—C2B	109.3 (11)
C16—C15—H15	120.7	C22B—C21B—H21C	109.8
C11—C16—C15	120.6 (3)	C2B—C21B—H21C	109.8
C11—C16—H16	119.7	C22B—C21B—H21D	109.8
C15—C16—H16	119.7	C2B—C21B—H21D	109.8
O141—N14—O142	123.8 (4)	H21C—C21B—H21D	108.3
O141—N14—C14	118.4 (4)	C21B—C22B—H22D	109.5
O142—N14—C14	117.8 (4)	C21B—C22B—H22E	109.5
C6A—C1A—C2A	119.6 (10)	H22D—C22B—H22E	109.5
C6A—C1A—N1	117.7 (7)	C21B—C22B—H22F	109.5
C2A—C1A—N1	122.2 (6)	H22D—C22B—H22F	109.5
C3A—C2A—C1A	116.8 (9)	H22E—C22B—H22F	109.5
C3A—C2A—C21A	120.0 (10)	C4B—C3B—C2B	121.3 (10)
C1A—C2A—C21A	118.9 (9)	C4B—C3B—H3B	119.4
C22A—C21A—C2A	112.5 (13)	C2B—C3B—H3B	119.4
C22A—C21A—H21A	109.1	C5B—C4B—C3B	120.4 (10)
C2A—C21A—H21A	109.1	C5B—C4B—H4B	119.8
C22A—C21A—H21B	109.1	C3B—C4B—H4B	119.8
C2A—C21A—H21B	109.1	C4B—C5B—C6B	119.3 (10)
H21A—C21A—H21B	107.8	C4B—C5B—H5B	120.4
C21A—C22A—H22A	109.5	C6B—C5B—H5B	120.4
C21A—C22A—H22B	109.5	C5B—C6B—H6B	119.8
			
C1A—N1—C17—C11	−178.1 (3)	C17—N1—C1A—C2A	−151.9 (12)
N1—C17—C11—C16	−177.7 (3)	C6A—C1A—C2A—C3A	−12 (6)
N1—C17—C11—C12	1.4 (5)	N1—C1A—C2A—C3A	178 (2)
C16—C11—C12—C13	−0.3 (5)	C6A—C1A—C2A—C21A	−168 (5)
C17—C11—C12—C13	−179.4 (3)	N1—C1A—C2A—C21A	21 (2)
C11—C12—C13—C14	0.0 (5)	C3A—C2A—C21A—C22A	97 (3)
C12—C13—C14—C15	0.3 (5)	C1A—C2A—C21A—C22A	−107 (2)
C12—C13—C14—N14	179.0 (3)	C1A—C2A—C3A—C4A	7 (5)
C13—C14—C15—C16	−0.4 (5)	C21A—C2A—C3A—C4A	163 (3)
N14—C14—C15—C16	−179.1 (3)	C2A—C3A—C4A—C5A	3 (7)
C12—C11—C16—C15	0.2 (5)	C3A—C4A—C5A—C6A	−7 (9)
C17—C11—C16—C15	179.4 (3)	C2A—C1A—C6A—C5A	7 (11)
C14—C15—C16—C11	0.1 (5)	N1—C1A—C6A—C5A	179 (6)
C15—C14—N14—O141	171.1 (4)	C4A—C5A—C6A—C1A	2 (12)
C13—C14—N14—O141	−7.7 (5)	C3B—C2B—C21B—C22B	12 (4)
C15—C14—N14—O142	−8.9 (5)	C21B—C2B—C3B—C4B	−162 (4)
C13—C14—N14—O142	172.3 (4)	C2B—C3B—C4B—C5B	−1 (7)
C17—N1—C1A—C6A	37 (5)	C3B—C4B—C5B—C6B	5 (10)

### Hydrogen-bond geometry (Å, º)

**Table:**

*D*—H···*A*	*D*—H	H···*A*	*D*···*A*	*D*—H···*A*
C16—H16···O141^i^	0.93	2.54	3.456 (5)	167

Symmetry code: (i) −*x*+3/2, −*y*+1, *z*+1/2.
